# Advancing laboratory medicine in hospitals through health information exchange: a survey of specialist physicians in Canada

**DOI:** 10.1186/s12911-020-1061-z

**Published:** 2020-02-28

**Authors:** Louis Raymond, Éric Maillet, Marie-Claude Trudel, Josianne Marsan, Ana Ortiz de Guinea, Guy Paré

**Affiliations:** 1 Université du Québec à Trois-Rivières, Trois-Rivières, Canada; 2 Université de Sherbrooke, Sherbrooke, Canada; 3 HEC Montréal, Montreal, Canada; 4 Université Laval, Quebec City, Canada; 5 Research Chair in Digital Health, HEC Montréal, 3000, Côte-Sainte-Catherine Road, Montréal, Québec H3T 2A7 Canada

**Keywords:** Laboratory information exchange, Information systems, Laboratory medicine, Specialist physician, Hospital, Perceived benefits, Online survey research

## Abstract

**Background:**

Laboratory testing occupies a prominent place in health care. Information technology systems have the potential to empower laboratory experts and to enhance the interpretation of test results in order to better support physicians in their quest for better and safer patient care. This study sought to develop a better understanding of which laboratory information exchange (LIE) systems and features specialist physicians are using in hospital settings to consult their patients’ laboratory test results, and what benefit they derive from such use.

**Methods:**

As part of a broader research program on the use of health information exchange systems for laboratory medicine in Quebec, Canada, this study was designed as on online survey. Our sample is composed of 566 specialist physicians working in hospital settings, out of the 1512 physicians who responded to the survey (response rate of 17%). Respondents are representative of the targeted population of specialist physicians in terms of gender, age and hospital location.

**Results:**

We first observed that 80% of the surveyed physicians used the province-wide interoperable electronic health records (iEHR) system and 93% used a laboratory results viewer (LRV) to consult laboratory test results and most (72%) use both systems to retrieve lab results. Next, our findings reveal important differences in the capabilities available in each type of system and in the use of these capabilities. Third, there are differences in the nature of the perceived benefits obtained from the use of each of these two systems. Last, the extent of use of an LRV is strongly influenced by the IT artefact itself (i.e., the hospital’s LRV available capabilities) while the use of the provincial iEHR system is influenced by its organizational context (i.e. the hospital’s size and location).

**Conclusions:**

The main contribution of this study lies in its insights into the role played by context in shaping physicians’ choices about which laboratory information exchange systems to adopt and which features to use, and the different perceptions they have about benefits arising from such use. One related implication for practice is that success of LIE initiatives should not be solely assessed with basic usage statistics.

## Background

Laboratory testing occupies a prominent place in health care [1]. For instance, more than 7 billion laboratory tests are performed each year in the United States [2]. It is also reported that about 70% of all medical decisions are based on laboratory test results [3]. In hospital settings, which are the focus of the present study, 98% of admitted patients have one or more laboratory tests prescribed [4]. To provide services across a broad continuum and to perform increasingly complex tests, laboratories require sophisticated medical technologies and highly qualified staff [1]. Faced with this growing complexity, treating physicians must be able to rely on consistent clinical support provided by laboratory medicine specialists, much like radiologists and pathologists [5, 6].

A recent study found that among seven countries, Canada ranked second in terms of physician self-reported errors in laboratory and diagnostic processes, as well as delays in reporting abnormal results [7]. One way to improve the quality and safety of patient care is to emphasize prevention and error management using well-designed information technology (IT) systems [8, 9]. Indeed, the laboratory testing process involves the constant exchange of information among patients, physicians, nurses, and laboratory specialists which, nowadays, is supported by multiple IT systems and platforms [10].

Missing laboratory results may have considerable consequences for patients and are due to several factors: (1) the systems and practices used to monitor test results, (2) the management of critical results, and (3) care transitions across settings [10]. To prevent medical errors [8], medical laboratories have deployed laboratory information systems (LIS) with user-friendly interfaces, e-tracking tools and electronic alerts [5, 11], computerized physician order entry (CPOE), and clinical decision support capabilities [12]. These systems empower laboratory specialists to enhance the interpretation of test results in order to better support physicians in their quest for better and safer patient care [5]. Although physicians may have access to LIS, these systems are primarily designed to meet the needs of laboratory personnel. Therefore, other laboratory information exchange (LIE) systems are required to improve the reliability of the laboratory testing process [13] and, hence, need to be integrated with other clinical information systems physicians use in hospitals such as electronic health records (EHRs) [14, 15].

Prior research in the information systems (IS) field draws two main conclusions that are pertinent to this study. First, the mere adoption of a given IT system is not enough to achieve improvements in performance [16]. In fact, prior investigations of the relationship between IT system use (i.e., duration or frequency of use) and individual and organizational performance outcomes have yielded contradictory and inconclusive results (e.g., [17–19]). Instead, it appears that performance improvements depend more on *how* a given IT system is used than on for how long [20, 21]. More precisely, recent research shows that the *extended use* of a given IT system (i.e., conceptualized as the extent to which system features are utilized) is positively related to performance outcomes [22]. Research in the medical informatics field has recently corroborated the relationship between extended use of a system features and performance outcomes such as quality of care, efficiency, operational performance, and economic performance [23]. Second, the IS literature has, for the most part, failed to conceptualize the IT artifact *objectively* [20]. That is, instead of capturing the features available in a system, researchers have focused on mental representations of the system (e.g., perceived ease of use, perceived usefulness) [24, 25]. Such mental representations are not of practical use, as they do not give any information about how the capabilities available in a system shape its extended use, nor do they provide concrete feedback to system designers about the criticality of certain features or the need for additional ones. As a result, IT systems, such as LIE, need to be better conceptualized in terms of their key functionalities or features.

Considering the above, the present study pursues two main objectives. First, it sought to develop a better understanding of which LIE systems and features specialist physicians (SPs) working in hospital settings are using to consult their patients’ laboratory test results, and what benefits they derive from such usage. More precisely, we attempt to provide answers to the following research questions: What is the nature of LIE usage in hospitals, and what types of information systems and features are being used by SPs for laboratory medicine purposes? How extensive is this use? What are the benefits obtained by SPs from extended LIE usage? Second, this study attempts to identify the contextual factors that lead to or influence the extended use of LIE systems by SPs. While medical informatics researchers have investigated the facilitators and barriers related to the adoption of EHRs in hospital settings (e.g., [26–29]), to our knowledge no prior study has focused on the antecedents to LIE system usage per se. The present study attempts to fill this gap. As explained below, inspired by prior research on EHRs we investigated the individual, organizational and IT artefactual antecedents to LIE use.

## Methods

**Fig. 1:**
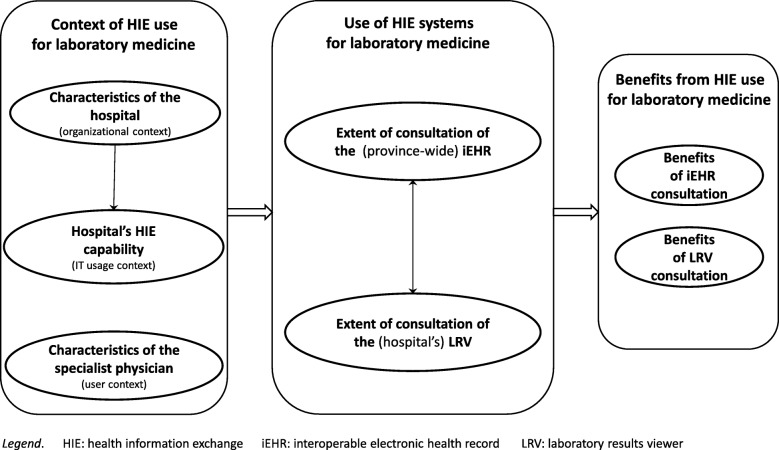
Conceptual Framework

As shown in Fig. 1, a conceptual framework was developed to describe and explain SPs’ use of health information exchange (HIE) systems for laboratory medicine in hospital settings, as well as the potential antecedents and performance outcomes of such use. This framework guided the design of the survey administered to find answers to our research questions. The framework is founded on prior research on HIE use in hospital settings and on the impacts of such use on laboratory testing in particular (e.g., [5, 15, 29, 30]). Moreover, we followed Burton-Jones and Grange [16] in assuming that using HIE systems per se do not necessarily enable laboratory medicine in hospital care. Our conceptual framework thus implies that only an “extended” use of LIE systems can have a positive impact on the practice of laboratory medicine by SPs, in terms of their efficiency and the quality of the care services provided to their patients [23].

As part of a broader research program on the use of HIE systems for laboratory medicine in the province of Quebec, Canada, this study was designed as an online survey. As described below, we followed best practices concerning web-based survey methodology [31]. The survey questionnaire was built following the previously mentioned review of the extant literature and a series of interviews with 25 physicians located in 11 different regions of Quebec. Survey respondents were recruited with the help of the Quebec’s Ministry of Health and Social Services, which emailed an invitation letter to the 9005 physicians who had authorized access to the province-wide interoperable electronic health record (iEHR), called the Quebec Health Record (QHR). The letter included a hyperlink and a QR code for mobile devices, directing respondents to access the survey questionnaire through a secure Web page. Developed with the Qualtrics online survey platform [32], the survey instrument was first approved by the province’s health authorities and then pre-tested with 10 physicians. Each physician was interviewed about the questionnaire’s format and instructions, as well as the wording of questions and possible answers, to ensure that they were interpreted as intended by the researchers. Following a few minor adjustments to the survey instrument, the study received final approval from the ethics committee of each researcher’s institution. Two reminder letters were sent to all targeted physicians 7 and 14 days after the initial invitation.

Our sample is composed of 566 SPs providing secondary or tertiary care in hospital settings, out of the 1512 physicians who fully responded to the survey (for a 17% response rate). The potential for non-response bias was ascertained by comparing the 112 “late” respondents (i.e. those who answered after receiving the second reminder) with the 454 “early” respondents. No significant differences were found between these two groups, thus indicating the absence of such a bias. The data were then analyzed through descriptive statistics, Chi-squared analysis, structural equation modeling (using SmartPLS software), cluster analysis and analysis of variance and covariance (using SPSS software). The internal validity of the two index measures of HIE use was ascertained with “item analysis”, in which we confirmed that each measure correlated sufficiently with its component items [33]. The internal validity of the two scale measures of the impacts of HIE systems use was tested with Cronbach’s α coefficient (> 0.6 threshold for exploratory research).

## Results

As shown in Table 1 (see top section), 49% of the SPs in our sample were women. In terms of clinical experience, 34% had less than 10 years of experience, 35% had 10 to 24 years, and 31% had 25 years or more. All major medical specialties are represented, including psychiatry, anesthesiology, pediatrics, radiology, internal medicine, surgery, obstetrics-gynecology, cardiology and others. Respondents were asked to indicate what their main work affiliation was and to describe their use of HIE systems in this context. All SPs practised in hospital settings; 44% in small or medium-size establishments (1 to 149 specialists) and 56% in large ones (150 or more specialists). As to their location, 70% practiced in a hospital located in a central or urban region, whereas 30% worked in peripheral or rural regions. It is worth noting that our respondents are representative of the targeted population of SPs in terms of gender (46% are women), age (average is 49 years old) and location (65% work in hospitals located in central or urban regions).^1^

**Table 1:** Context of HIE use by specialist physicians (SPs) for laboratory medicine

**Characteristics of the SPs** (user context)	**All specialist physicians**	**iEHR**^a^ **users**	**iEHR non-users**	**Chi-squared statistic**
	(*N* = 566) freq. (%)	(*n* = 451) freq. (%)	(*n* = 115) freq. (%)	
Gender				
Female	278 (49.1)	221 (49.0)	57 (49.6)	0.0
Male	288 (50.9)	230 (51.0)	58 (50.4)	
Age				
29 years old or less	7 (1.2)	6 (1.3)	1 (0.9)	5.6
30–49 years old	317 (56.0)	256 (56.8)	61 (53.1)	
50–59 years old	137 (24.2)	101 (22.4)	36 (31.3)	
60 years old or more	105 (18.6)	88 (19.5)	17 (14.8)	
Clinical experience				
5 years or less	85 (15.0)	72 (16.0)	13 (11.3)	5.9
5–9 years	107 (18.9)	85 (18.8)	22 (19.1)	
10–24 years	196 (34.6)	156 (34.6)	40 (34.8)	
25 years or more	178 (31.4)	138 (30.6)	40 (34.8)	
**Characteristics of the SPs’ hospital facility **(organizational context)	**All specialist physicians**	**iEHR**^a^ **users**	**iEHR non-users**	**Chi-squared statistic**
	(*N* = 566) freq. (%)	(*n* = 451) freq. (%)	(*n* = 115) freq. (%)	
Size of the hospital				
1-49specialist physicians	110 (19.4)	84 (18.6)	26 (22.6)	1.1
50–149 specialist physicians	139 (24.6)	111 (24.6)	28 (24.3)	
150–299 specialist physicians	170 (30.0)	136 (30.2)	34 (29.6)	
300–1500 specialist physicians	147 (26.0)	12 (26.6)	27 (23.5)	
Location of the hospital				
Urban region	395 (69.8)	340 (75.4)	55 (47.8)	33.0***
Rural region	171 (30.2)	111 (24.6)	60 (52.2)	
**HIE systems used by the SPs for laboratory medicine** (IT artefactual context)	**All specialist physicians**	**iEHR**^a^ **users**	**iEHR non-users**	**Chi-squared statistic**
	(*N* = 566) freq. (%)	(*n* = 451) freq. (%)	(*n* = 115) freq. (%)	
Laboratory Results Viewer^b^				
LRV non-user	42 (7.4)	42 (9.3)	0 (0.0)	11.6***
LRV user	524 (92.6)	409 (90.7)	115 (100)	

^a^Quebec Health Record

^b^For viewing lab results provided by a LIS, a CIS, and/or a regional HIE platform

* | *** The χ^2^ value indicates a significant difference (*p* < 0.05 | *p* < 0.001) between iEHR users and non-users

In terms of the health IT artefacts used for HIE purposes, Table 1 reveals that 451 (80%) SPs consult laboratory test results through the province-wide iEHR and 524 (93%) through a LRV. In simple terms, an LRV is a common interface that allows physicians to access test results from their hospital’s clinical information system (CIS), a public or private medical laboratory’s information system (LIS), and/or their region’s HIE platform (RHIEP)^2^ [34, 35]. Despite being labelled “viewer”, some LRVs also have CPOE features, thus allowing laboratory tests prescription. The iEHR^3^ is deployed by the Quebec government within the context of Canada’s national healthcare system [36]. It appears that significantly more of the SPs who do not use the province-wide iEHR practice medicine in rural regions.

Table 2 presents the different types of HIE systems used by the surveyed physicians. In this regard, there appears to be three main HIE use cases, a first case in which a SP uses only the iEHR, a second case in which he or she uses only a LRV, and a third case in which both types of HIE systems are used in combination. The third case is the most prevalent, as a large majority of the sampled SPs (72%) are found to retrieve lab results through both the iEHR and an LRV. However, it is noteworthy that the SPs may use an LRV but not the iEHR to order new lab tests. Conversely, only 28% of the SPs in our sample use a single source to retrieve lab test results, either the iEHR (8%) or an LRV (20%). Moreover, the SPs’ use of an LRV is quite varied in terms of the combination of systems (LIS, CIS and RHIEP) that they access for lab purposes through the common interface provided by the hospital. For instance, 32% of LRV users access laboratory test results through both their hospital’s CIS and their regional HIE platform.

**Table 2:** Types of HIE systems used by specialist physicians for laboratory medicine

**HIE use case**	**HIE system used**	**Number of specialist physicians**
I	(iEHR)	42 (7.4%)
II	(LRV)	115 (20.3%)
III	(iEHR) & (LRV)	409 (72.3%)
Total		566 (100%)
**LRV use case**	**HIE system used**	**Number of specialist physicians**
i	(CIS)	149 (28.4%)
ii	(LIS)	55 (10.5%)
iii	(RHIEP)	20 (3.8%)
iv	(CIS) & (LIS)	66 (12.6%)
v	(CIS) & (RHIEP)	170 (32.4%)
vi	(LIS) & (RHIEP)	10 (1.9%)
viii	(CIS) & (LIS) & (RHIEP)	54 (10.3%)
Total		524 (100%)

*Legend*. *iEHR* interoperable electronic health record, *LRV* laboratory results viewer, *CIS* clinical information system, *LIS* laboratory information system, *RHIEP* regional HIE platform

Table 3 reveals important differences in the HIE capabilities available in each type of system, LRV and iEHR, and in the actual use of these capabilities by SPs. For instance, the possibility of electronically requesting a laboratory analysis and printing identifying labels for the samples is a capability that is available in only 55% of the LRV systems consulted by SPs. Yet 48% of the SPs are using it, leaving only 7% of the SPs with access to the functionality not using it. However, SPs seem to use most of the HIE capabilities available to them, utilizing on average 81 and 77% of the consultation capabilities available in the iEHR and their LRV, respectively. A notable exception is that only 39% of LRV users access patients’ test results produced by the laboratories in their region, even though this capability is available in 89% of the LRV systems.

**Table 3:** HIE consultation capabilities for laboratory medicine used by specialist physicians

**HIE capability for laboratory medicine**	**Availability of HIE capability **(% of systems)	**Use of HIE capability** (% of SPs)
LRV consultation capabilities for laboratory medicine (*n* = 524)		
*The laboratory results viewer allows me to …*		
• access all laboratory test results of a patient, whether I prescribed such tests or not;	92.9%	91.8%
• only access those patients’ test results that are produced by my hospital’s laboratory;	95.0%	87.6%
• generate tables and graphs for the display and analysis of lab test results;	82.5%	79.4%
• apply search criteria in order to find the lab test results that meet my needs;	79.6%	78.7%
• access patients’ test results that are produced by the laboratories in my region;	88.5%	38.8%
• electronically request a laboratory analysis and print identifying labels for the samples.	55.3%	48.3%
**iEHR consultation capabilities for laboratory medicine** (*n* = 451)		
*I consult the laboratory results provided by the QHR*^*a*^ *when …*		
• the patient has been seen by a physician in another health establishment in Quebec	100%	98.9%
• the patient is unable to reliably report to me his or her recent laboratory test results or his or her present state of health;	100%	98.4%
• the patient’s laboratory test results that I require are not found in my usual information sources (clinical information system – CIS, paper reports, etc.);	100%	98.2%
• the patient has no medical record in the hospital;	100%	95.8%
• doing an outpatient clinical consultation;	100%	93.8%
• I do not know the patient;	100%	80.0%
• I am consulted for a patient under observation in the emergency room;	100%	78.3%
• I am consulted for an admitted patient at the hospitalization unit;	100%	76.0%
• I am consulted in relation to a surgical intervention, a treatment or a diagnostic exam (e.g. in the operating room, in oncology);	100%	63.9%
• a physician beckons my expertise and I must take note of the clinical case remotely.	100%	60.3%

^a^Quebec Health Record

The next set of results pertains to the performance outcomes of HIE use in hospitals for laboratory testing, i.e. to the benefits perceived by SPs in terms of their individual efficiency and the quality of the care provided to their patients. As indicated in Table 4, there are important differences in the nature of the benefits obtained from each of the two types of systems used by SPs and in the extent to which these benefits were realized. For LRV users, the most important benefits were the greater, quicker and easier access to lab test results. For users of the province-wide iEHR platform, the most critical benefits for their practice include significant improvements in continuity of care and in their ability to make better clinical decisions.

**Table 4:** Perceived benefits from specialist physicians’ use of HIE for laboratory medicine

**Benefits of HIE use for laboratory medicine**^a^	**Mean (s.d.)**
Benefits of LRV consultation (*n* = 524)	
• It is quicker for me to access the viewer to consult patients’ previous lab test results than wait to receive their paper medical record	4.2 (1.1)
• My patients’ lab test results are easier to consult in the viewer than in the paper medical record	4.1 (0.9)
• The viewer provides most of the lab test results that I need to care for patients referred to me	4.0 (1.1)
• As compared with the paper medical record, the follow-up of admitted patients’ lab test results when changing the patient surveillance team is made easier with the viewer	3.8 (1.2)
• As most of my patients reside in the region, I have little use for the QHR^b^ because the viewer provides me with most of the lab test results that I need	2.7 (1.4)
• The viewer is very useful in allowing me to access test results produced by the public laboratories in my region	2.6 (1.6)
**Benefits of iEHR consultation** (*n* = 451)	
*Accessing the laboratory test results provided by the QHR*	
• improves the continuity of my patients’ care	4.2 (0.8)
• provides me with results that I cannot obtain from my usual information sources (e.g. lab results viewer)	4.1 (0.8)
• allows me to make better clinical decisions	4.0 (0.8)
• improves the way in which I evaluate patients	3.8 (0.9)
• has reduced the duplication of the lab tests prescribed to my patients	3.8 (1.0)
• prevents me from missing an important result;	3.7 (1.0)
• increases the safety of my patients’ care;	3.7 (0.9)
• allows me to intervene more rapidly and effectively with my patients;	3.6 (0.9)
• provides me with an overall view of my patients’ lab results (patients’ test history)	3.6 (1.0)
• allows me to ask for more advanced lab analyses whose results may be useful to other clinicians	3.6 (1.0)
• provides support to my clinical research or my performance measurement activities	1.6 (1.0)

^a^As perceived by the specialist physicians on Likert scales of 1 (strongly disagree) to 5 (strongly agree)

^b^Quebec Health Record

**Fig. 2:**
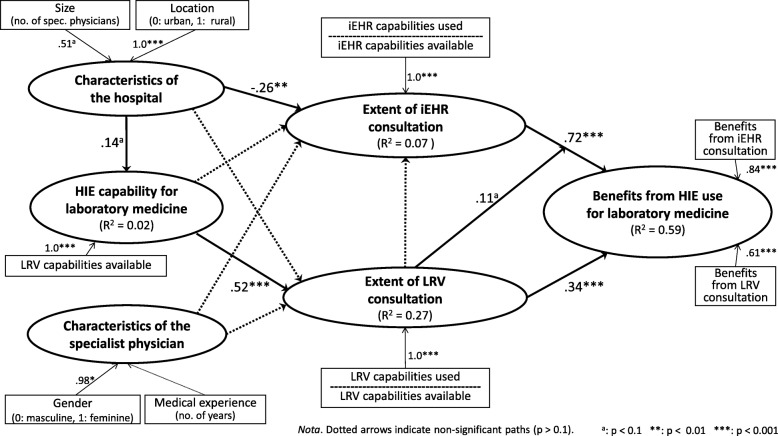
Path analysis of the use of HIE for laboratory medicine in hospitals (PLS, n = 566)

Component-based structural equation modeling (SEM) was used to explore empirically the causal paths implied by our research framework. The partial least squares (PLS) method was thus selected because it is better suited to measurement models such as ours that include both exogenous and endogenous “formative” constructs [37], as presented in Fig. 2. As implemented in the SmartPLS software, this technique was also chosen for its robustness in terms of the distribution of residuals and its greater affinity for exploratory rather than confirmatory research purposes when compared to covariance-based SEM methods [38].

The first step consisted of simultaneously estimating the measurement and structural models using PLS. Psychometric properties of construct indicators (measures) were thus assessed, noting that the measurement model includes only formative constructs. Given that the usual reliability and validity criteria, such as composite reliability and average variance extracted, do not apply to formative constructs, it must first be verified that there is no multicollinearity among the indicators forming such constructs [39]. This was verified with the variance inflation factor (VIF), based on the guideline that this statistic should be smaller than 3.3 for any formative indicator [40].^4^ As shown in Table 5, this condition held for all indicators. The last property to be verified is discriminant validity, which shows the extent to which each construct in the research model is unique and different from the others. The discriminant validity of a formative construct is demonstrated by a correlation with any other construct that is significantly different from unity (at *p* < 0.001) [41]. Such validity is confirmed here, as the highest correlation between any two of the six research constructs is 0.65 (between “Extent of iEHR use” and “Benefits from HIE use”).

**Table 5:** Descriptive statistics, reliability and inter-correlation of the variables

Variable (*n* = 566)	mean	s.d.	α^a^	VIF^b^	1.	2.	3.	4.	5.	6.	7.	8.
Characteristics of the hospital												
1. Size (no. of SPs)	220	246	–	1.10	–							
2. Location (0: urban, 1: rural)	.30	.46	–	1.10	−.30	–						
HIE capability for lab. Medicine												
3. LRV capabilities available	3.3	1.7	–	–	.05	.12	–					
Characteristics of the spec. Physician												
4. Gender (0: male, 1: female)	.49	–	–	1.04	−.05	.08	.04	–				
5. Medical experience^c^	3.8	1.9	–	1.04	.14	−.21	−.01	−.20	–			
Extent of LRV consultation												
6. LRV capab. Used / LRV capab. Available	.75	.34	–	–	.06	.02	.52	.08	−.02	–		
Extent of iEHR consultation												
7. iEHR capab. Used / iEHR capab. Available	.61	.34	–	–	.02	−.26	−.06	−.01	−.05	−.01	–	
Benefits from HIE consultation												
8. Benefits from LRV consultation	3.3	1.0	.62	1.01	−.26	.19	.63	.08	−.10	.59	−.13	–
9. Benefits from iEHR consultation	3.1	1.2	.85	1.01	.02	−.23	−.06	.04	−.05	.04	.86	−.08

^a^Cronbach’s reliability coefficient [inappropriate for index variables]

^b^Variance inflation factor

^c^1 = less than 5 years, 2 = 5–9 years, 3 = 10–14 years, 4 = 15–19 years, 5 = 20–24 years, 6 = 25 years or more

The causal paths were tested by assessing the path coefficients (β) estimated by the SEM procedure executed by the SmartPLS software. The performance of the structural model is assessed by the strength and significance of the path coefficients and the proportion of explained variance, as befits PLS’s focus on prediction and concern with generalization [42]. Returning to Fig. 2, a first result of note is the positive and highly significant path coefficients linking the extensive consultation of an LRV (β = 0.34, *p* < 0.001) and of the province-wide iEHR (β = 0.72, p < 0.001) to the attainment of benefits from HIE for laboratory medicine. This empirically confirms our initial assumption that HIE use by SPs must be “extended” if these physicians are to become more efficient and improve quality of care through such use.^5^ Furthermore, while the extent of the SPs’ consultation of an LRV is uncorrelated to the extent of their consultation of the iEHR (*r* = − 0.01), these two types of use do in fact interact, albeit rather weakly, as shown by the moderating effect of LRV use on the relationship between iEHR use and the benefits of HIE use (β = 0.11, *p* < 0.1). Therefore, the beneficial impact of extended consultation of the iEHR by the SPs appears to be enhanced when this use is combined with extended consultation of their hospital’s LRV.

Another result worth noting is that the extent of the SPs’ consultation of an LRV is essentially determined by their hospital’s HIE capability, or more specifically by the number of consultation capabilities available in their LRV, as indicated by a positive and highly significant path coefficient (β = 0.52, *p* < 0.001). This last result confirms that some SPs have more consultation capabilities than others, depending upon the hospital setting. It is important to note, however, that this argument does not concern the province-wide iEHR system, as it provides all physicians with the same consultation capabilities for laboratory medicine, independent of the hospital setting. In fact, the LRV capability available to SPs is uncorrelated to the extent of their consultation of the iEHR (*r* = − 0.06).

While the extent of consultation of an LRV is strongly influenced by the IT usage context (i.e. the hospital’s LRV capability), the extent of consultation of the iEHR is rather influenced by its organizational context (i.e. the hospital’s size and location). More precisely, a negative and significant path coefficient indicates that this consultation is more extended in hospitals that tend to be smaller and located in urban regions (β = − 0.26, *p* < 0.01). This may be related to the fact that organizational context was also found to influence the IT usage context, albeit weakly. More specifically, the LRV capability is stronger in hospitals that tend to be larger and located in rural regions, as indicated by a positive and significant path coefficient (β = 0.14, *p* < 0.1). Finally, one must note that, contrary to what was expected, the SPs’ individual characteristics in terms of gender and medical experience did not play a significant role in determining the extent to which they use HIE for laboratory medicine purposes. Moreover, the organizational, IT artefactual and user characteristics that constitute the context of HIE systems use were found to explain a significantly greater percentage of variance in the physicians’ extent of LRV consultation (27%) than in their extent of iEHR consultation (7%).

To generate added insight and provide further explanations of the use of HIE for laboratory medicine in hospital settings, we took an alternative approach to further analyze our survey data. As opposed to the preceding “causal” approach, we used a “configurational” approach which makes no assumptions as to the statistical distribution of the research variables, nor as to the linearity of the relationships between these variables [43]. As operationalized with methods such as cluster analysis, this approach is meant to provide a more-encompassing, holistic view of the use of HIE by SPs for laboratory medicine purposes. A cluster analysis was thus used to group our survey respondents into HIE usage profiles, such that each profile’s membership was homogeneous in terms of HIE systems use. The SPSS Two-Step clustering algorithm was chosen, as it can handle many cases, automatically determines the optimal number of clusters (profiles) and has been found to be the top-performing clustering algorithm [44].

A three-cluster solution was found to be optimal, i.e. the most interpretable and meaningful in identifying HIE usage profiles that could be clearly distinguished from one another. The high quality of the clusters in terms of cluster compactness and separation was confirmed by a silhouette measure [45]. As shown in Table 6, the 367 SPs (65%) in the first profile were named *LRV-iEHR-reliant* users, as they were found to make extensive use of the capabilities for laboratory medicine available in both an LRV and the iEHR system. A second group of 119 SPs (21%) were named *LRV-reliant* users, as they extensively consulted an LRV but their consultation of the iEHR was very limited or null. Last, the third HIE usage profile, named *iEHR-reliant*, consists of 80 SPs (14%) who consulted the iEHR extensively but whose consultation of an LRV was very limited.

**Table 6:** Profile analysis of the use of HIE systems for laboratory medicine in hospitals

**Characterization of the specialist physicians’** **use of HIE for laboratory medicine**	**HIE usage profiles**			ANOVA F	ANCOVA^¶^F
	*LRV-iEHR-reliant* *users* (*n* = 367) mean	*LRV-* *reliant users* (*n* = 119) mean	*iEHR-reliant users* (*n* = 80) mean		
**Extent of HIE consultation**^a^					
Extent of consultation of the hospital’s LRV	0.89_1_	0.76_2_	0.05_3_	642***	397***
Extent of consultation of the (province-wide) iEHR	0.77_1_	0.01_2_	0.77_1_	1628***	1497***
**HIE capability of the hospital**					
No. of LRV consultation capabilities available	3.8_1_	3.6_1_	0.9_2_	153***	-
**Characteristics of the hospital**					
Size (no. of specialist physicians)	232	214	181	1.6	-
Location [0: urban, 1: rural]	0.25_2_	0.51_1_	0.25_2_	16.7***	-
**Characteristics of the user**					
Gender [0: male, 1: female]	0.51	0.50	0.49	1.2	-
Clinical experience^b^	3.7	3.9	4.1	2.1	-
**Benefits from HIE use for laboratory medicine**^c^					
Benefits of LRV consultation	3.6_1_	3.6_1_	1.8_2_	191***	61***
Benefits of iEHR consultation	3.6_1_	1.0_2_	3.4_1_	1063***	978***

*Nota*. Within rows, different subscripts indicate significant (*p* < 0.05) pair-wise differences between means (Tamhane’s T2 test)

^¶^With five control variables: HIE capability, characteristics of the hospital, characteristics of the user

^a^Clustering variables [no. consultation capabilities used / no. of consultation capabilities available] ***p < 0.001

^b^[1 = 5 years or less, 2 = 5-9, 3 = 10-14, 4 = 15-19, 5 = 20-24, 6 = 25 years or more]

^c^As perceived by the specialist physician on Likert scales of 1 [strongly disagree] to 5 [strongly agree]

To identify individual, organizational and IT artefactual antecedents to HIE usage by SPs, we sought to contextualize the three HIE usage profiles that emerged from our analyses. As shown in Table 6, the three user groups do not differ significantly in terms of individual characteristics, i.e. gender and medical experience. In terms of organizational context, the *LRV-iEHR-reliant* and *iEHR-reliant* users work in hospitals located in urban regions, as opposed to the *LRV-reliant* users, more of whom practice in a rural region. The IT usage context is defined by a hospital’s HIE capability, more specifically the number of consultation capabilities available to SPs within each HIE system used for laboratory medicine purposes. Unsurprisingly, all three user groups have access to the same HIE capabilities from the province-wide iEHR platform. However, *LRV-iEHR-reliant* and *LRV-reliant* users perceive their LRV to include significantly more HIE capabilities than *iEHR-reliant* users who, for the most part, do not use an LRV. Thus, except for the province-wide iEHR, other systems such as the CIS, LIS and RHIEP consulted by the SPs through an LRV appear to differ in terms of the number of laboratory consultation capabilities that are made available to SPs. Such differences in HIE capability may explain why SPs differ in the extent to which they consult these systems in their daily practice.

Our last set of findings pertains to differences in performance outcomes of LIE usage among the three user groups. Returning to Table 6, one finds that the first group, the *iEHR-LRV-reliant* users, receive as many benefits from their consultation of the province-wide iEHR system as the *iEHR-reliant* users, whereas the *LRV-reliant* users receive very limited benefits from this system. Recalling that the most important benefits of iEHR consultation pertained to the quality of care provided by the SPs to their hospitalized patients, whereas the main benefits of LRV consultation pertained instead to physician efficiency, it appears that the *iEHR-LRV-reliant* physicians obtain, on average, the highest performance outcomes for all aspects. Such differences in performance outcomes among the three groups would thus be mainly explained by differences in HIE capability, i.e. by the number of HIE capabilities that are actually available to SPs, and by the extent to which HIE systems such as an LRV and the iEHR are actually consulted by physicians.

## Discussion

While the findings of this study confirm that benefits are derived from using HIE for laboratory medicine, they demonstrate that these impacts are not the same for every physician. Here follow some tentative explanations for the variations observed. First, considering the IT ecosystem used to access laboratory test results, we observed many combinations of systems. Interestingly, SPs who work in rural regions tend to use the province-wide iEHR less than those who work in urban hospitals. These *LRV-reliant* users contrast with the *LRV-iEHR-reliant* and *iEHR-reliant* users, who mainly work in hospitals located in urban regions. This could indicate that the government provides less service support for the iEHR in more peripheral areas in Quebec. It could also reflect the service trajectory of patients seen by SPs practicing in rural areas. For instance, it is possible that these specialists mainly see patients from their own region, patients for whom all the laboratory results are included in their local CIS, accessible via an LRV. In contrast, large tertiary care hospitals located in urban centers are more likely to treat patients from other regions and SPs in these settings most likely access the iEHR to consult laboratory results. However, when treating local patients, lab results can be accessed from an LRV. This is precisely the profile found in Table 6 for *LRV-iEHR-reliant* users. Finally, the *iEHR-reliant* users are mainly working in small hospitals located in urban centers. We can hypothesize that these are small community health centers with fewer resources than large university hospitals or institutes and are not able to invest in the deployment of sophisticated LRV systems. In such cases, it is likely that the iEHR became accessible while they were still working with paper trails, making any spending on such a system useless after the arrival of the iEHR. In fact, as presented in Table 7, a post hoc analysis of variance performed on our data set uncovered that *iEHR-reliant* users tended to use more paper than *LRV-reliant* or *LRV-iEHR-reliant* users. This finding corroborates our insights.

**Table 7:** Profile analysis of the use of paper for laboratory medicine in hospitals

Characterization of the specialist physicians’ use of paper for laboratory medicine	HIE usage profiles			ANOVA F
	*LRV-iEHR-reliant users* (*n* = 367) mean	*LRV-reliant users* (*n* = 119) mean	*iEHR-reliant users* (*n* = 80) mean	
Use of paper (0: no, 1: yes)	0.06	0.08	0.15	3.5*

*p < 0.05

Second, regarding the functionalities available to and used by SPs, we observed important differences in terms of what was available and what was used. We also realized that, despite their availability, most functionalities were not used by an average of 3.9% of the surveyed physicians. One intriguing result is that although 88.5% of the SPs mentioned that their LRV allowed them to access patients’ test results from several laboratories in their region, only 38.8% mentioned they were in fact using this capability, a difference of 49.7%. One possible explanation for this could draw on the specificity of practicing specialist medicine. In the urgency to act often linked to the intervention of a SP, patients may have their laboratory tests performed in the same institution where they met their specialist, eliminating the need for the SP to access results from other regional laboratories. Conversely, it may also simply be a standard procedure for SPs to have laboratory tests being systematically redone. These factors, along with others, should be explored more carefully in future research. We can contrast these findings with those of prior studies about the use of laboratory-related capabilities in electronic health record (EHR) systems. In recent studies, while a vast majority of family physicians mentioned that their EHR system allowed them to view laboratory results and used such functionality, other core capabilities such as electronic ordering and tracking of laboratory tests were found to be much less available in these systems and, hence, much less used (e.g., [23, 46]).

SEM was used to explore the causal paths in our research framework. It was found that while the extent of the SPs’ consultation of an LRV was mainly determined by the number of capabilities these systems offer,^6^ the extent of the SPs’ consultation of the iEHR was rather influenced by the organizational context. To this effect, it was found that iEHR consultation was less extended in large hospitals as well as those located in rural regions. A tentative explanation could be that large regional hospitals are the first places where people go for specialized care. Specialists in these hospitals have long needed access to results from laboratories outside their own institution yet located in their region. Considering the many delays experienced in the deployment of the provincial iEHR project [47], several regions have chosen to organize around an RHIEP to achieve these results. As seen previously, this capability adds to the more basic ones of visualizing the results of their LIS or CIS, thus explaining the positive coefficient between the characteristics and the HIE capabilities, as well as the negative coefficient between the characteristics and the extent of iEHR usage, since it does not really offer any additional benefits. Thus, for SPs practicing in large regional hospitals, the main reason for using the iEHR would be that they must treat patients from other regions, a task that usually behooves to specialists in urban centers.

Lastly, it was found that SPs did not perceive that the same benefits were obtained from using the iEHR and an LRV. Considering that only 80 (14%) SPs in our sample primarily use the iEHR, it can be assumed that most of them have access to an LRV. Hence for most respondents, the benefits of using the iEHR supplement the benefits of using their viewer. It thus seems normal for items related to continuity of care and better clinical decision-making to score higher than items related to ease and speed of access to laboratory results. This is reinforced by the lack of correlation between the availability of LRV capabilities and the extent of consultation of the iEHR, demonstrating that these are complementary systems, not equivalent ones.

Of the findings discussed so far, all point to one main observation: the use of a system is first motivated by a need, which is in turn influenced by context. For SPs, this need largely revolves around where the treated patients come from, since this is what determines, first and foremost, where laboratory test results can be retrieved. In this light, one important implication of this study is that large iEHR initiatives, such as the QHR project in Quebec, should not be assessed with basic usage statistics, especially when what is valued is the number of adopters.

## Conclusions

The main purpose of this study was to develop a better understanding of which LIE systems and features SPs are using in hospitals to consult their patients’ laboratory test results and what benefits they derive from such use. Our research has the limitations generally associated with survey research. First, its response rate was 17%. Second, although some survey questions relate to facts, most relate to perceptions, and this may induce bias in the results. The organizational context stood out as an important element determining the extent of the SPs’ use of different IT systems for consulting laboratory results. However, as the hospital’s size and location explained only a small percentage of variance in the extent of HIE use by SPs, other organizational characteristics and in particular the hospital’s status (e.g., general/secondary care/ vs. specialized/tertiary care, non-affiliated vs. university-affiliated hospital) should be accounted for in future research. The same could be said of user characteristics, where the SPs’ gender and medical experience should be complemented by such characteristics as their computer literacy and HIE experience. Moreover, future research should collect and analyze data about patient trajectories to deepen our understanding of the reasons behind the nature of HIE systems used for laboratory medicine purposes. Another way to assess this would be to conduct in-depth case studies of how and why SPs use these systems. This would allow us to better understand how different contexts lead to different uses of HIE.

The main contribution of this study to theory lies in its insights into the role played by context in shaping SPs’ choices about which HIE systems to use and which features of these systems to use, and the different perceptions they have about benefits arising from such use. Our findings resonate with Davison and Martinsons [48], who mention how “[e]ach phenomenon or case is based on a distinctive context even as it has certain general properties.” (p. 242). Therefore, future research on HIE systems should explicitly conceptualize the key contextual dimensions of the study at the outset of theorization. Our analysis supports the need for examining contextual considerations when studying IT systems [49].

From a practical standpoint, our findings show that, contrary to what was expected, the SPs’ individual characteristics examined in this study do not play a significant role in determining the extent to which they use HIE for laboratory medicine purposes. This means that there is no need to develop recruitment strategies based on individual characteristics if the objective is to ensure extensive use of HIE for laboratory medicine. Moreover, our study sheds light on the complementary nature of iEHRs and LRVs. There may exist different profiles of features complementarity depending on the context of use. From a design standpoint, system designers should take a step back to imagine a way to design systems as part of an interconnected network of features, which is what a HIE should be. That is, they should at the outset of their endeavor, consider the complementarity of the system in development to the whole ecosystem of IT artefacts. In the same vein, system vendors should reflect on the place of their own systems in this ecosystem and provide specific training about the complementary use of their system features. This could certainly help SPs to better understand and extensively use HIE systems and derive all possible benefits for themselves and their patients, and hence overcoming the ceiling effect in HIE assimilation [50].

## Supplementary information

**Additional file 1.** Questionnaire survey items.

## Data Availability

The datasets used and/or analyzed during this study are available from the corresponding author on reasonable request.

## Abbreviations

CIS: Clinical information system
CPOE: Computerized physician order entry
EHR: Electronic health record
HIE: Health information exchange
iEHR: Interoperable electronic health record
IS: Information system
IT: Information technology
LIE: Laboratory information exchange
LRV: Laboratory results viewer
PLS: Partial least squares
QHR: Quebec health record
RHIEP: Regional health information exchange platform
SEM: Structural equation modeling
SP: Specialist physician
VIF: Variance inflation factor

## Footnotes

1 Source: https://www.fmsq.org/en/profession/repartition-des-effectifs-medicaux.

2 A RHIEP is a multi-sided platform. On the one side, hospitals which usually include laboratories along with major CIS systems, join the platform and upload their patients’ data to the RHIEP’s database. On the other side, physicians query RHIEP’s database and download the available laboratory information.

3 The iEHR system deployed in Quebec, called the Quebec Health Record or QHR, is a secure provincial tool that is used to collect, store and release information about patients’ health. It is organized into 3 clinical domains: medications, laboratories and medical imaging. The health information contained in the QHR can be released on request to authorized providers and bodies in accordance with their access authorization. For more information: https://www.quebec.ca/en/health/your-health-information/quebec-health-record/.

4 VIF_*i*_ = 1/(1-R_*i*_^2^), where R_*i*_^2^ is the unadjusted R^2^ obtained when component_*i*_ is regressed against all other components of the formative construct.

5 This is consistent with other studies on the use of EMR systems in primary clinics. The extent to which the EMR is used by family physicians positively and significantly influences their own perceptions in terms of performance benefits [e.g., 23].

6 It is worth mentioning that this result is consistent with other studies conducted in primary care clinics which found that the EMR’s functional coverage positively and significantly influences the extent of EMR use by family physicians [23].
