# Supplementary material for: Advancing laboratory medicine in hospitals through health information exchange: a survey of specialist physicians in Canada
Source: BMC Med Inform Decis Mak. 2020 Feb 28;20:44. doi: 10.1186/s12911-020-1061-z (PMC7048105; doi:10.1186/s12911-020-1061-z)
Supplement: Supplementary file 1 — Additional file 1. Questionnaire survey items. [file 12911_2020_1061_MOESM1_ESM.docx]

**Appendix. Questionnaire survey items**

Individual and hospital profiles:

What is your gender?

O Male

O Female

What is your age group?

O 29 years old or less

O 30 - 49 years old

O 50 - 59 years old

O 60 years old or more

How much experience do you have as a specialist physician?

O 5 years or less

O 5 - 9 years

O 10 - 24 years

O 25 years or more

What is your main specialty?

O Allergy and immunology

O Anesthesiology

O Dermatology

O Emergency medicine

O Internal medicine

O …

What is the size of the hospital you work at? (approximate number of specialist physicians)

O 1-49

O 50-149

O 150-299

O 300-1,500

Where is the hospital you work at located?

O Urban or central region

O Rural or peripheric region

Use of HIE for laboratory medicine purposes:

Which of the following types of systems do you use on a regular basis to access your patients’ laboratory test results? (*check all that apply*)

O The interoperable electronic health record (Quebec Health Record)

O A laboratory results viewer (LRV)

O The clinical information system available at your hospital (e.g., Oacis, Ariane, Cristal-Net, Dossier patient Purkinje, Helios)

O A laboratory information system (e.g., Softlab, Meditech, Cerner, Calculus, Omnilab, Technidata, QuadraMed)

O A regional platform (e.g., MediResult/MediClinic)

HIE capabilities for laboratory medicine:

Are the following capabilities available in the LRV and, if so, do you use them?

| **HIE capability for laboratory medicine** | **Available in LRV** | **I do…** |
| --- | --- | --- |
| - access all laboratory test results of a patient, whether I prescribed such tests or not | Yes/No | Yes/No |
| - only access those patients’ test results that are produced by my hospital’s laboratory | Yes/No | Yes/No |
| - generate tables and graphs for the display and analysis of lab test results | Yes/No | Yes/No |
| - apply search criteria in order to find the lab test results that meet my needs | Yes/No | Yes/No |
| - access patients’ test results that are produced by the laboratories in my region | Yes/No | Yes/No |
| - electronically request a laboratory analysis and print identifying labels for the samples | Yes/No | Yes/No |

In which circumstances do you consult/access laboratory tests results in the QHR system?

| - the patient has been seen by a physician in another health establishment in Quebec | Yes/No |
| --- | --- |
| - the patient is unable to reliably report to me his or her recent laboratory test results or his or her present state of health | Yes/No |
| - the patient’s laboratory test results that I require are not found in my usual information sources (clinical information system – CIS, paper reports, etc.) | Yes/No |
| - the patient has no medical record in the hospital | Yes/No |
| - doing an outpatient clinical consultation | Yes/No |
| - I do not know the patient | Yes/No |
| - I am consulted for a patient under observation in the emergency room | Yes/No |
| - I am consulted for an admitted patient at the hospitalization unit | Yes/No |
| - I am consulted in relation to a surgical intervention, a treatment or a diagnostic exam (e.g. in the operating room, in oncology) | Yes/No |
| - a physician beckons my expertise and I must take note of the clinical case remotely | Yes/No |

Perceived benefits from specialist physicians’ use of HIE for laboratory medicine:

| *Benefits associated with consulting lab results in the LRV* | 1 | 2 | 3 | 4 | 5 |
| --- | --- | --- | --- | --- | --- |
| - It is quicker for me to access the viewer to consult patients’ previous lab test results than wait to receive their paper medical record |  |  |  |  |  |
| - My patients’ lab test results are easier to consult in the viewer than in the paper medical record |  |  |  |  |  |
| - The viewer provides most of the lab test results that I need to care for patients referred to me |  |  |  |  |  |
| - As compared with the paper medical record, the follow-up of admitted patients’ lab test results when changing the patient surveillance team is made easier with the viewer |  |  |  |  |  |
| - As most of my patients reside in the region, I have little use for the QHR^b^ because the viewer provides me with most of the lab test results that I need |  |  |  |  |  |
| - The viewer is very useful in allowing me to access test results produced by the public laboratories in my region |  |  |  |  |  |
| 1=strongly disagree 2=disagree; 3=undecided; 4=agree; 5=strongly agree | | | | | |

| *Consulting lab results in the provincial QHR…* | 1 | 2 | 3 | 4 | 5 |
| --- | --- | --- | --- | --- | --- |
| - Improves the continuity of my patients’ care |  |  |  |  |  |
| - Provides me with results that I cannot obtain from my usual information sources (e.g. lab results viewer) |  |  |  |  |  |
| - Allows me to make better clinical decisions |  |  |  |  |  |
| - Improves the way in which I evaluate patients |  |  |  |  |  |
| - Has reduced the duplication of the lab tests prescribed to my patients |  |  |  |  |  |
| - Prevents me from missing an important result |  |  |  |  |  |
| - Increases the safety of my patients’ care |  |  |  |  |  |
| - Allows me to intervene more rapidly and effectively with my patients |  |  |  |  |  |
| - Provides me with an overall view of my patients’ lab results (patients’ test history) |  |  |  |  |  |
| - Allows me to ask for more advanced lab analyses whose results may be useful to other clinicians |  |  |  |  |  |
| - Provides support to my clinical research or my performance measurement activities |  |  |  |  |  |
| 1=strongly disagree 2=disagree; 3=undecided; 4=agree; 5=strongly agree | | | | | |
